# Effects of Lutein and Docosahexaenoic Acid Supplementation on Macular Pigment Optical Density in a Randomized Controlled Trial

**DOI:** 10.3390/nu5020543

**Published:** 2013-02-15

**Authors:** Alfredo García-Layana, Sergio Recalde, Angel Salinas Alamán, Patricia Fernández Robredo

**Affiliations:** Ophthalmology Department, Clinica Universidad de Navarra, C/ Pio XII 36, 31080, Pamplona, Spain; E-Mails: srecalde@unav.es (S.R.); asalinas@unav.es (A.S.A.); pfrobredo@unav.es (P.F.R.)

**Keywords:** lutein, docosahexaenoic acid, age-related macular degeneration, macular pigment density

## Abstract

We studied the macular pigment ocular density (MPOD) in patients with early age macular degeneration (AMD) before and 1 year after nutritional supplementation with lutein and docosahexaenoic acid (DHA). Forty-four patients with AMD were randomly divided into two groups that received placebo (*n* = 21) or a nutritional supplement (*n* = 23, 12 mg of lutein and 280 mg of DHA daily). Heterochromatic flicker photometry was used to determine the MPOD. At baseline, the MPOD in AMD patients with placebo was 0.286 ± 0.017 meanwhile in AMD patients with supplementation it was 0.291 ± 0.016. One year later, the mean MPOD had increased by 0.059 in the placebo group and by 0.162 in patients receiving lutein and DHA. This difference between groups was significant (*p* < 0.05). Lutein and DHA supplementation is effective in increasing the MPOD and may aid in prevention of age related macular degeneration.

## 1. Introduction

Understanding the pathogenesis of age-related macular degeneration (AMD), the most common cause of visual disability in elderly patients in developed countries, is advancing rapidly, but is still unclear [1,2]. Epidemiologic reports have suggested that diets rich in vitamins C and E, zinc, lutein, zeaxanthin, and docosahexaenoic acid (DHA) are associated with the greatest reduction in the risk of development of early and advanced AMD [3,4]. Higher dietary intake of lutein and zeaxanthin was associated independently with a decreased likelihood of having AMD [5] and dietary omega-3 long-chain polyunsaturated fatty acid intake is associated with a decreased risk of progression from bilateral drusen to advanced AMD [6]. Lutein, zeaxanthin and meso-zeaxanthin are xanthophylls (carotenoids that contain one or more polar functional groups) that selectively accumulate in the retina and are particularly dense in the foveal region, or macula, where they are the main components of the macular pigment (MP) [7]. In addition, lutein is the precursor of meso-zeaxanthin, a major component of MP [8]. They are known to function as antioxidants [9] and blue-light filters and thereby may protect the macular retina and retinal pigment epithelium from light-initiated oxidative damage [10]. Oxidative stress is high in the eye because of repeated exposure to light and the high rate of oxidative metabolism in the retina. In addition, the LAST [11] and LUNA [12] studies have evaluated the beneficial effect of lutein supplementation in patients with AMD.

DHA is a fatty acid found in the retina, with a high concentration in the rod outer segment [13,14]. Because photoreceptor outer segments are constantly being renewed, a constant supply of DHA may be required for proper retinal function. Marginal depletion may impair retinal function and influence the development of AMD [15]. Moreover, in two prospective follow-up studies it was reported that DHA intake was inversely related to the risk of AMD [3,16]. Of note is the observation that supplemental DHA increases HDL and HDL subfractions in serum [17,18,19]. Given that the carotenoids transport is made by lipids (HDL) [20,21], DHA supplementation could increase its transport to the retina and subsequently cause a MP ocular density (MPOD) increase. Therefore, DHA may, in part, decrease risk of AMD via increased transport of lutein into the macula. MPOD has been evaluated as a predictor of the retinal response to nutritional intervention with lutein in patients with AMD with the hope of retarding visual loss, disease progression, or both [22].

Although the formulation in the AREDS, which prevents development of advanced AMD, was comprised of vitamins C and E, beta-carotene, and zinc, currents trends in AMD nutritional prevention also are based on the use of lutein and long-chain polyunsaturated fatty acids like DHA [23].

Few data have been reported on the effect of the combined supplementation in MPOD [15]. Johnson *et al.* [15] reported that although lutein supplementation increases MPOD in nonsmoking elderly women without AMD, when lutein and DHA were administered together, the increase in MPOD was not significant. This study investigates the influence of lutein and DHA supplementation on MPOD and vision performance.

## 2. Experimental Section

### 2.1. Sample Size and Inclusion Criteria

For reasons aforementioned, we conducted a small prospective study to determine the basal MPOD in 44 patients with early AMD (stage II-III AREDS classification corresponding to small/intermediate drusen and large drusen with/without pigment changes). All subjects underwent a screening examination that included a medical history and a physical examination. Volunteers with any history of lactose intolerance, liver, kidney, or pancreatic disease, anemia, insulin-dependent diabetes, hyperlipoproteinemia or alcoholism were excluded from the study. Other exclusion criteria included current use of antihistamine drugs, steroids or nonsteroidal anti-inflammatory drugs and use of any nutrient supplement for previous 2 months or carotenoid supplements for the previous 6 months. The present project has been performed following the Declaration of Helsinki, was approved by the Ethics Committee of the Clinica Universidad de Navarra and all participants received and signed an informed consent for the study. Randomization was done by coin toss by the same ophthalmologist who enrolled them in the study. 

### 2.2. Nutritional Supplementation

The patients with AMD were distributed randomly in two groups; one group (placebo group) were asked to take two placebo tablets daily for one year (*n* = 21), and the other group (intervention group) were asked to take two tablets daily of a supplemental complex with lutein and DHA (*n* = 23). The two intervention tablets contained a total daily dosage of 12 mg of lutein, 0.6 mg of zeaxanthin, and 280 mg of DHA. The placebo (containing sugar) and intervention tablets presented with the same look, smell, taste, packaging, and were manufactured by the same laboratory (Laboratorios Thea, Barcelona, Spain). Patients as well as ophthalmologists were blinded as to which group were taking the placebo tablets and which group were taking the intervention tablets until the end of the study.

### 2.3. Macular Pigment Ocular Density (MPOD) Measurement

The instrument used was the Eye Maculometer^®^ (modified version: [24], School of Biosciences, University of Westminster, London, UK) to provide central fixation for both the foveal and parafoveal condition. This device is based on Heterochromatic Flicker Photometry (HFP) and uses Light Emitting Diodes (LED) as the near monochromatic light source, similarly to other published studies for the assessment of MPOD [22,24,25,26,27]. The Eye Maculometer^®^ described in 1998 had only one test field and required eccentric fixation for the parafoveal measurement. This field was imaged on the fovea by direct fixation by the subject or on a patch of retina 5 degrees from the fovea by getting the subject to fixate on a small red light placed to one side of the single test field. Many subjects found this eccentric fixation was not easy to maintain. Consequently, the Eye Maculometer^®^ was modified to provide central fixation for both the foveal and parafoveal condition. This improved device was the used in our study [24]. 

### 2.4. Visual Parameters

Visual function was tested before and after supplementation by measuring the best-corrected visual acuity with the ETDRS chart (Vectorvision, Greenville, Ohio, USA), and by contrast sensitivity with Pelli-Robson charts (Clement Clarke International, Edinburgh Way, Harlow, Essex, UK). Macular thickness was measured by Stratus optical coherence tomography (Carl Zeiss Meditec, Jena, Germany).

### 2.5. Statistical Analysis

Values are reported as the mean ± standard error of the mean (SEM)*.* Statistical significance was evaluated by T student or U Mann-Whitney tests to analyze the differences between the placebo group and the nutritional supplementation group. Statistical significance was accepted at the 95% confidence level (*p* < 0.05) and analysis was performed by using the computer program SPSS (version 15.0, SPSS Inc., Chicago, IL, USA).

## 3. Results

### 3.1. Participant Characteristics

Of 69 participants screened for this study, 44 were AMD patients that were assigned randomly to receive treatment or placebo. Of the 44 participants, 26 (59.0%) were women and 18 (41.0%) were men. Mean age ± Standard Error of Mean (S.E.M.) was 67.8 ± 9.2 years for AMD patients with placebo and 69.2 ± 7.8 years for AMD patients with supplementation. Mean body mass index ± S.E.M. was 24.8 ± 1.4 kg/m^2^ for placebo group and 25.2 ± 1.5 kg/m^2^ for supplementation group. There were not statistical differences between both groups in any of these demographic parameters (Table 1).

**Table 1:** General Parameters and basal macular pigment ocular density (MPOD) in age macular degeneration (AMD) patients with placebo and supplementation with lutein and docosahexaenoic acid (DHA).

	Placebo group (*n* = 21)	Intervention group (*n* = 23)
Men/women	8/13	10/13
Age (years)	67.8 (9.2)	69.2 (7.8)
BMI (kg/m^2^)	24.8 (1.4)	25.2 (1.5)
MPOD^ a^	0.286 (0.017)	0.291 (0.016)

BMI = body mass index. Age, BMI, and MPOD measurements are expressed as the mean ± S.E.M. ^a^ *p* < 0.01.

### 3.2. Macular Pigment Optical Density (MPOD)

**Figure 1:**
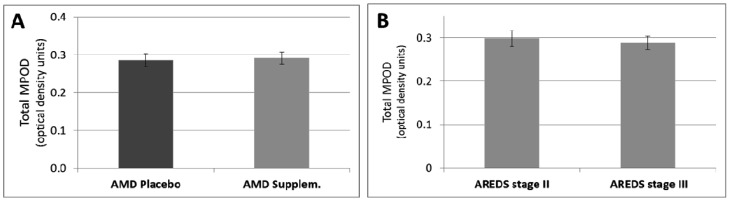
(**A**) MPOD in AMD patients with placebo and AMD patients with supplementation. (**B**) MPOD separately for the Age Related Eye Disease study (AREDS) categories 2 and 3. Data are presented as means ±S.E.M.

The baseline MPOD in the intervention group was (0.291 ± 0.016 unit) and was not statistically different from that of the placebo group (0.286 ± 0.017 unit) (*p* > 0.05, Table 1, Figure 1A). Additionally, there was no significant difference between MPOD in the AREDS categories 2 and 3 (*p* > 0.05, Figure 1B).

**Figure 2:**
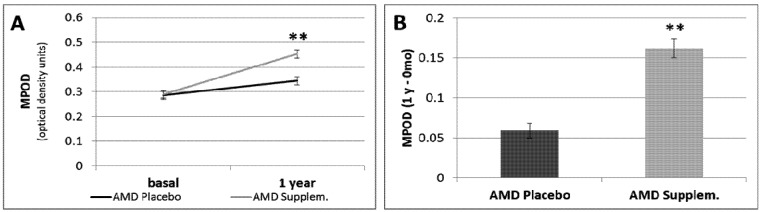
(**A**) MPOD in AMD placebo group and AMD supplemented group at time 0 (basal) and one year after treatment. (**B**) The MPOD change (1 year–0 month) in AMD placebo group and AMD supplemented group. Data are presented as means ±S.E.M. ** *p* <0.01.

When we compared the MPOD values in patients with AMD after 1 year of follow-up, we found a significant increase in the supplementation group (0.453 ± 0.028 unit) compared to the placebo group (0.345 ± 0.026 unit) (*p* < 0.01, Table 2, Figure 2A). Moreover, total MPOD increased very significantly after nutritional supplementation for 1 year (*p* < 0.01; Figure 2B). However, the VA, contrast sensitivity, and macular thickness did not change after 1 year of supplementation (Table 2). In addition, the increase (0.059 units) in MPOD in the group taking placebo after 1 year compared to baseline was not statistically significant. When we evaluated the non-responders to supplementation, *i.e.*, patients with less than a 5% increase in the total MPOD, four of 23 were non-responders among the patients with AMD who received nutritional supplement (17.4%).

**Table 2:** Ophthalmic parameters in patients with early AMD who received placebo or supplementation with lutein and DHA.

Parameters	Period	*Placebo Group (n* *=* *21)*	*Intervention Group (n* *=* *23)*	*P Value*
MPOD	*Baseline*	0.286 (0.017)	0.291 (0.016)	
	*1 year after*	0.345 (0.026)	0.453 (0.028)	*p* < 0.01
ETDRS (letters)	*Baseline*	78.3 (6.2)	76.4 (8.7)	
	*1 year after*	75.9 (5.8)	74.3 (9.2)	ns
Contrast sensitivity score (letters)	*Baseline*	26 (5)	25(5)	
	*1 year after*	26 (6)	26 (5)	ns
OCT macular thickness (μm)	*Baseline*	246 (20.7)	248 (32.5)	
	*1 year after*	249 (29.8)	246 (43.2)	ns

BCVA = best-corrected visual acuity; OCT = optical coherence tomography. All values are expressed as the mean (± S.E.M.). The Best Corrected Visual Acuity tested with the Early Treatment Diabetic Retinopathy Study charts and contrast sensitivity are expressed in number of letters (ETDRS). *p* < 0.01.

## 4. Discussion

This study investigates the influence of lutein and DHA supplementation on MPOD and vision performance. We found a significant increase in the MPOD levels in patients with AMD treated for 1 year with nutritional supplementation compared with the patients with AMD who received placebo, indicating that supplemental pills with lutein and DHA can significantly improve MP and increase retinal antioxidant protection in patients with this retinal disease. These results also agree with other similar studies and reaffirmed the hypothesis that the lutein and zeaxanthin macular concentrations constitute a modifiable nutritional risk factor [28]. However, visual function did not improve after supplementation, a result also reported previously [29]. Of note is that the increase in MPOD in the group taking placebo was not statistically significant. Our hypothesis is that those patients suffered the “placebo effect” and their behavior on dietetics could have changed. Probably, they were more motivated and took better care of themselves. 

Our results differed from those of Johnson *et al.* [15] who found that the MPOD did not differ significantly when lutein and DHA were administered together. However, there are several differences between both populations that may explain that difference. Firstly, the population in the study of Johnson *et al.* was comprised of only women, and the current study included both genders. Secondly and probably more important, the population in the study of Johnson were patients without AMD and our supplementation was given to patients with AMD, who may have a better response to that supplementation, because those individuals with the lowest MPOD values were in the greatest need of supplementation and the most likely to benefit [15]. 

Additionally, the percentages of non-responders to lutein and DHA supplementation in our study were even lower than those observed by other authors in AMD patients (17.4% and 21.4%) [15]. In contrast, several recent studies in normal subjects [25] and healthy subjects with atypical spatial profile of MP (lack of a typical central peak) [27] showed a low rate of non-responders (<5%) because of the rapid increase in MP by the meso-zeaxanthin supplementation. They suggested that family history of AMD and smoking cigarettes may inhibit meso-zeaxanthin generation from lutein at the macula [25,27]. This hypothesis could explain the high rate of non-responder in our older AMD population supplemented with lutein given that it is well documented that atypical spatial profiles are more common in older subjects like AMD patients [26]. Moreover, it is possible that the non-responders in supplementation studies are individuals who lack the ability to convert lutein into meso-zeaxanthin. However, recent data has shown that when meso-zeaxanthin is provided in a supplement, it has a rapid and dramatic effect on serum carotenoid levels [27,30]. Thus, in our non-responders meso-zeaxanthin supplementation could be more effective than lutein. 

Our device is based on the subjective HFP method, however several limitation need to be taken into account. For example: The inability to customise the flicker and the fact that this works in an independent mode as opposed to the yoked mode. However, we have found similar results than other authors using objective techniques [28]. Moreover, another limitation was the small sample size and the use of a subjective technique such as HFP. Therefore, the results obtained herein should be interpreted with full appreciation of their sample size and the device. This fact could result in a likelihood of chance findings in either direction. Thus, it would be necessary to complete the study with more information with respect to diet, smoking, exercise, plasma lutein levels, *etc*.

## 5. Conclusions

In conclusion, the daily intake of a nutritional complex containing 12 mg of lutein, 0.6 mg of zeaxanthin, and 280 mg of DHA had a beneficial effect on the MPOD levels in patients with AMD. However further clinical trials like AREDS 2 [31] are required to investigate the optimum dosage levels of all vitamins, micronutrients, and carotenoids that will protect the retina against degenerative diseases such as AMD.
